# Supplementary material for: Complex‐centric proteome profiling by SEC‐SWATH‐MS
Source: Mol Syst Biol. 2019 Jan 14;15(1):e8438. doi: 10.15252/msb.20188438 (PMC6346213; doi:10.15252/msb.20188438)
Supplement: Supplementary file 6 — Dataset EV5 [file MSB-15-e8438-s006.zip › feature_plots_corum/5380.pdf]

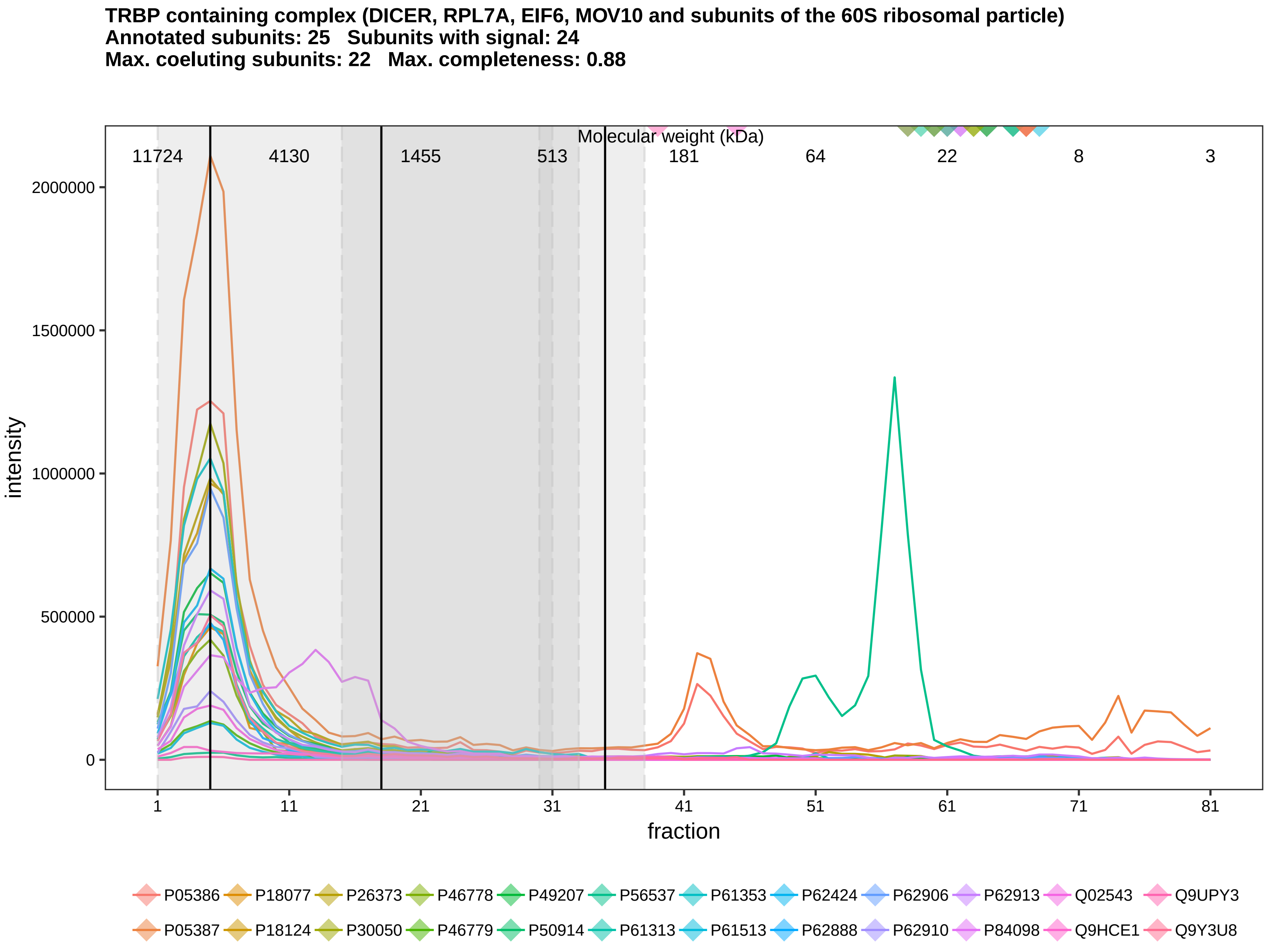

TRBP containing complex (DICER, RPL7A, EIF6, MOV10 and subunits of the 60S ribosomal particle)
Annotated subunits: 25 Subunits with signal: 24
Max. coeluting subunits: 22 Max. completeness: 0.88
Molecular weight (kDa)
11724
4130
1455
513
181
64
22
8
3
2000000
1500000
1000000
500000
0
intensity
1
11
21
31
41
51
61
71
81
fraction
P05386
P05387
P18077
P18124
P26373
P30050
P46778
P46779
P49207
P50914
P56537
P61313
P61353
P61513
P62424
P62888
P62906
P62910
P62913
P84098
Q02543
Q9HCE1
Q9UPY3
Q9Y3U8
